# Twisted fiber microfluidics: a cutting-edge approach to 3D spiral devices

**DOI:** 10.1038/s41378-023-00642-9

**Published:** 2024-01-22

**Authors:** Shunsuke Kato, Daniel W. Carlson, Amy Q. Shen, Yuanyuan Guo

**Affiliations:** 1 Department of Electrical, Information and Physics Engineering, School of Engineering, Tohoku University, Aoba-ku, Sendai, 980-8579 Miyagi Japan; 2 Micro/Bio/Nanofluidics Unit, Okinawa Institute of Science and Technology, Onna, Kunigami-gun, 904-0495 Okinawa Japan; 3 Frontier Research Institute for Interdisciplinary Sciences (FRIS), Tohoku University, Aoba-ku, Sendai, 980-0845 Miyagi Japan; 4 Graduate School of Biomedical Engineering, Tohoku University, Aoba-ku, Sendai, 980-8579 Miyagi Japan; 5 Department of Physiology, Graduate School of Medicine, Tohoku University, Aoba-ku, Sendai, 980-8575 Miyagi Japan

**Keywords:** Nanoscience and technology, Engineering

## Abstract

The development of 3D spiral microfluidics has opened new avenues for leveraging inertial focusing to analyze small fluid volumes, thereby advancing research across chemical, physical, and biological disciplines. While traditional straight microchannels rely solely on inertial lift forces, the novel spiral geometry generates Dean drag forces, eliminating the necessity for external fields in fluid manipulation. Nevertheless, fabricating 3D spiral microfluidics remains a labor-intensive and costly endeavor, hindering its widespread adoption. Moreover, conventional lithographic methods primarily yield 2D planar devices, thereby limiting the selection of materials and geometrical configurations. To address these challenges, this work introduces a streamlined fabrication method for 3D spiral microfluidic devices, employing rotational force within a miniaturized thermal drawing process, termed as mini-rTDP. This innovation allows for rapid prototyping of twisted fiber-based microfluidics featuring versatility in material selection and heightened geometric intricacy. To validate the performance of these devices, we combined computational modeling with microtomographic particle image velocimetry (*μ*TPIV) to comprehensively characterize the 3D flow dynamics. Our results corroborate the presence of a steady secondary flow, underscoring the effectiveness of our approach. Our 3D spiral microfluidics platform paves the way for exploring intricate microflow dynamics, with promising applications in areas such as drug delivery, diagnostics, and lab-on-a-chip systems.

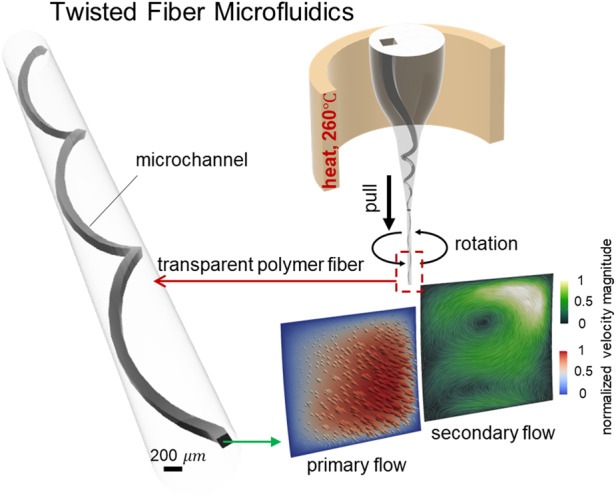

## Introduction

Microfluidics involves the science of manipulating and controlling small volumes of fluids, typically in the range of microliters to picoliters, within networks of channels having dimensions from tens to hundreds of micrometers. It serves as a versatile platform for a wide range of applications in life sciences, chemistry, materials science, and biotechnology. These applications include flow cytometry^1^, chemical reactions^2^, particle separation^3^, and mixing^4^. Mainstream microfluidic devices are primarily fabricated through photolithography or soft lithography on planar substrates. The range of materials compatible with these processes is relatively limited; for example, the elastomer poly(dimethylsiloxane) (PDMS) has been commonly used for the past two decades^5,6^. Furthermore, the geometric design of microfluidic channels often faces constraints due to limitations in processable cross-sectional shapes, such as squares or rectangles^6,7^.

One of the early pioneering works in continuous inertial focusing was conducted by Di Carlo’s group^8^. They exploited inertial lifting forces to manipulate particles within fluid flows effectively, achieving high throughput and efficiency with relatively simple setups^9,10^. However, conventional 2D planar microchannels, especially those with square or rectangular cross-sections, frequently exhibit multiple equilibrium points for particles. This phenomenon complicates the manipulation and control of particles within microfluidic systems, potentially leading to particle trapping or erratic behavior, thus affecting the performance and reliability of the devices.

To mitigate the presence of multiple equilibrium positions and enhance focusing efficiency, additional forces–either internal or external–such as electrical^11^ or magnetic fields^12^, can be introduced into the microfluidic flow. However, this approach often requires extra hardware, limiting the practicality and commercial viability of such systems. An alternative solution involves incorporating curvatures into the microfluidic device to induce centrifugal forces. In such cases, a secondary flow field, known as the Dean flow, is generated in proportion to the primary flow^13^. At high flow rates, these Dean vortices form at the cross-sections of the flow, improving particle repositioning efficiency.

Devices featuring such curvatures, particularly continuous spiral forms^14^, have found applications in n biomedicine, including cell sorting and particle focusing. Nevertheless, due to their inherent 2D planar structure, spiral microfluidic channels often experience channel radius expansion during flow. This leads to inconsistencies in the Dean flow as it moves through each spiral, making the prediction of equilibrium positions challenging^15^. Consequently, significant efforts have been made to innovate geometric and structural designs for spiral microfluidics, overcoming the limitations of traditional 2D planar configurations.

Microfluidic devices for practical applications typically undergo several prototyping iterations. These iterations demand rapid design and material modifications to achieve desired functional outcomes. Building complex 3D structures using traditional lithography techniques involves laborious and time-consuming procedures such as alignment, stacking, and bonding multiple layers^7,16^. Consequently, the low yield often falls short of meeting the needs for prototype evaluations, making the establishment of new manufacturing approaches essential for rapid and efficient 3D microfluidic prototyping.

In recent times, 3D printing has emerged as a widely accessible method for prototyping due to its ease of implementation and relatively low cost^17–22^. However, a significant drawback of 3D-printed microfluidic devices is their printing resolution, which is limited to tens of microns at minimum^18^. This limitation restricts the capability to design channels with dimensions down to the micrometer scale^18^. Additionally, the finished surfaces of 3D-printed devices often exhibit roughness, thereby limiting optical access for evaluating flow dynamics^6^.

Besides 3D printing, various studies have explored multi-step assembly processes to fabricate intricate 3D structures^5,7,16^. For example, Lim’s Group reported a straightforward extrusion and curing method for creating PDMS microtubes^5^. These microtubes were utilized as fundamental components for the assembly of 3D spiral microfluidic devices, achieved by coiling them around a fixed-radius rod. It is important to acknowledge that employing multi-step assembly processes presents challenges in scalability and material selection. Recent advancements in micromachining techniques, including femtosecond laser machining^23^ and abrasive jet machining^24^, have also been investigated for fabricating 3D spiral microfluidics. However, these techniques frequently involve complex, expensive, and time-consuming processes, rendering them unsuitable for the rapid prototyping of 3D spiral microfluidics.

A recent study by Yuan et al. delved into the use of a thermal drawing process–originally adapted from the telecommunications industry–to fabricate fibers incorporating microfluidics with diverse channel designs and a wide range of materials^25^. Their work primarily aimed to achieve inertial focusing using straight microfluidics within fibers and extended the design to include dielectrophoresis components for cell sorting. Nevertheless, the rapid prototyping of 3D spiral microfluidics, which could offer simple and efficient inertial focusing through Dean flow, remains an under-explored avenue with considerable potential for innovative microfluidic device development.

**Fig. 1:**
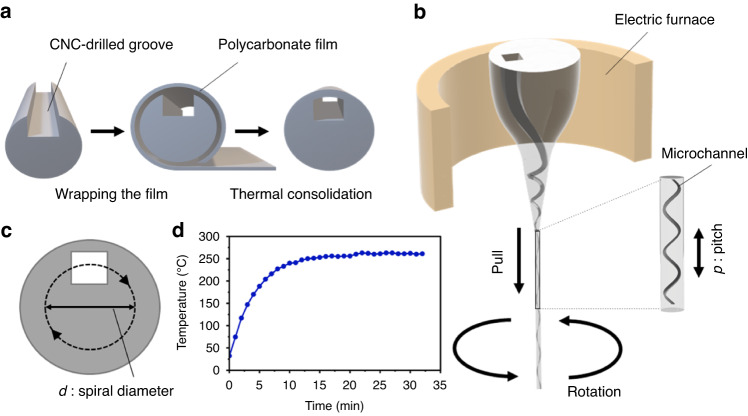
The fabrication procedure of creating 3D spiral fibers. **a** The schematics of the preform preparation process. **b** The illustration of the rotational thermal drawing process (rTDP). **c** The schematics of off-centered design allows for the generation of 3D spiral structures within the fiber during the rTDP. **d** The representative temperature characteristics of electric furnace used in rTDP, a key parameter in fiber spiral prototyping

In this work, we have developed a miniaturized fiber fabrication system by enhancing the traditional thermal drawing method with rotational capabilities. We refer to this novel method as mini-rTDP (miniaturized rotational thermal drawing process) (Fig. 1). The mini-rTDP technique enables the rapid prototyping and fabrication of flexible, polymer-based fibers that can reach lengths of several meters. Notably, these fibers feature twisted 3D spiral channels that resemble microscale parking ramps embedded within the fiber structure. When combined with CNC machining and 3D additive manufacturing techniques^26^, mini-rTDP facilitates the design and production of complex preforms, yielding fibers with tailored properties.

In this study, we used mini-rTDP to prototype fibers featuring diverse 3D microfluidic spirals efficiently. By designing single or multiple channels at off-center positions within the preform, key parameters governing the Dean flow–such as the spiral radius, pitch, and cross-sectional geometries–can be adequately controlled during the thermal drawing and rotation processes. We conducted detailed simulation studies to explore the flow dynamics within these 3D microfluidics. Additionally, we employed a novel experimental technique, tomographic particle image velocimetry (*μ*-TPIV), to visualize the 3D velocity field of the flow within the 3D spiral microfluidics. This method provided valuable insights into the underlying flow mechanisms within the fibers. Both the simulation and *μ*-TPIV results confirmed the successful generation of Dean vortices at high flow rates in the 3D spiral microfluidics within fibers, demonstrating the method’s efficiency for manipulating fluid dynamics and its potential for tuning particles’ equilibrium positions.

## Results and discussions

### Fabrication of twisted fibers microfluidics

To fabricate fibers with 3D spiral internal structures at the microscale, we prepared a preform made of polycarbonate (PC). This preform contains hollow channels with shapes that can be square, rectangular, or round, and dimensions at the millimeter scale, created through a standard macro-machining process. The preform serves as the foundational structure from which the fiber is drawn, using either thermal drawing or other fiber fabrication techniques. The choice of hollow channel shape within the preform depends on the desired internal structure of the resulting fiber (Fig. 1a). To introduce spiral patterns within the fiber during the thermal drawing process, we developed a mini-rTDP system, which is adapted from a previously developed miniaturized system^27^. In addition to the vertical pulling motion, we incorporated rotations in the horizontal plane to achieve the desired internal spiral structures within the fibers (Fig. 1b).

In the cross-sectional view of the fiber (Fig. 1c), the channels are not positioned along the centerline; instead, they are intentionally placed off-center. This means the channels are shifted away from the central axis of the fiber. Such off-center placement enables the generation of 3D spiral structures within the fiber during the thermal drawing process. The pitch *p* of the spiral is defined by *p* = *v*_*capstan*_/*v*_*rotation*_, where the *v*_*capstan*_ is measured in mm/s and *v*_*rotation*_ is given in rpm/60. This methodology correlates the spiral pitch with the drawing speed and the rotation speed, thus enabling precise control over the pitch of the spiral structures during the fiber fabrication process. By fine-tuning these parameters, one can achieve the desired pitch, thereby allowing for the customization and optimization of the resultant fiber’s internal spiral pattern.

### Characterization of microchannels within fibers

**Fig. 2:**
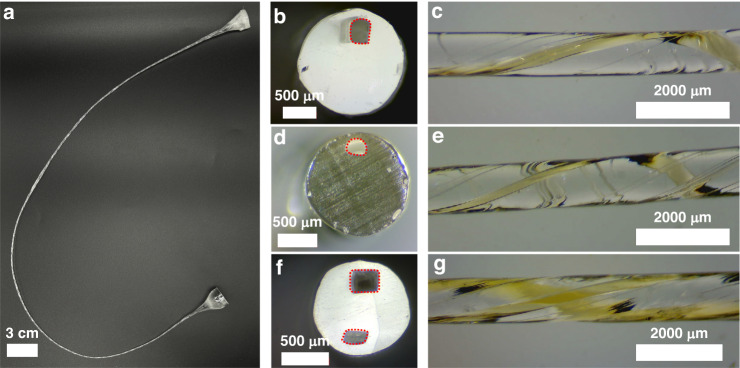
The prototyped fiber with internal spirals. **a** The photograph of the preform-to-fiber process, where fibers with tens of centimeters to meters can be produced in a simple and cost-efficient manner. **b** The cross-section of the fiber spiral with a square microchannel. The profile is contoured with a red dash line. **c** The side view shows the fiber spiral in **b**. **d** The cross-section of the fiber spiral with the rounded microchannel. The profile is contoured with a red dash line. **e** The side view shows the spiral turns of the fiber in **d**. **f** The cross-section profile of the fiber with double microchannels. Their profiles are contoured with red dash lines. **g** The side view of the fiber with double microchannels reveals parallel spirals

Through the utilization of the mini-rTDP system, we successfully fabricated a diverse array of 3D spiral fiber-based microfluidic devices. Figure 2b–g displays representative examples of microchannel cross-sections and 3D spiral structures. These results demonstrate versatility in channel geometry design, ranging from rectangular to semicircular and circular structures, as depicted in Fig. 2b (and Supplementary Fig. S3). The millimeter-scale channels present in the preform are scaled down to feature sizes of approximately 200–300 μm, with a typical drawn-down ratio of 20. This decrease in channel size results from the stretching and elongation that take place during the thermal drawing process. Our mini-rTDP system has also shown the capability to produce fibers with tunable spiral pitches, specifically those measuring 8.4 mm or greater. This broad range of achievable pitches enhances the flexibility in designing and functionalizing the microfluidic devices.

In addition, the geometry of the microchannels is preserved across the spirals, as shown in Supplementary Fig. S4. Moreover, the fabricated fiber devices made of PC are highly transparent, providing excellent optical access for visualizing flow dynamics. This clarity is particularly advantageous for imaging techniques, as the surface of the PC exhibits minimal interaction with the polystyrene (PS)-based fluorescent particles employed in our flow dynamics evaluations.

The inherent versatility of the preform-to-fiber drawing process enables us to incorporate multiple channels within the preform. These are subsequently transformed into a thin strand of fiber featuring multi-spiral microfluidics. This ability to integrate multiple channels into a single fiber introduces an additional layer of complexity and functionality to the microfluidic devices. Figure 2f presents an exemplary instance of multi-microchannels formed within a fiber, showcasing varying cross-sectional profiles and spiral radii. This design offers the significant advantage of enabling straightforward parallelization of microfluidics within a single fiber. Such parallelization facilitates the simultaneous and independent manipulation of diverse fluids, samples, or particles within each microchannel, thereby expanding the device’s capabilities and enhancing throughput.

However, it is imperative to note that two primary forces exert a significant influence on the channel profile during the rTDP process (Supplementary Fig. S5). The first is thermal reflow, a crucial factor both in consolidation and in the rTDP process, especially when dealing with microchannel profiles like squares or rectangles. This phenomenon arises due to Laplace pressure, a pressure differential formed between the internal and external regions of two fluid areas (Fig. 2d and Supplementary Fig. S6a). Sharp corners and edges of square and rectangular profiles tend to be rounded off into semi-circular forms due to the surface tension forces acting on the material during thermal processes. Dang et al. previously developed a model to clarify how feature sizes are conserved throughout the thermal drawing process. Their research highlights reflow time (*τ*) as a key parameter in this context:

1 $$\tau =\eta \lambda /\pi \gamma .$$

Here, the zero-shear viscosity (*η*) and the surface tension (*γ*) of the polymer solution are temperature-dependent, while *λ* represents the feature profile of the surface, i.e., the channel width (*D*_*h*_) in our case. To maintain the channel profile, one strategy is to maximize the characteristic reflow time, thereby minimizing the influence of thermal reflow. Several avenues exist for achieving this. One approach is to increase zero-shear viscosity *η* by selecting polymers known for superior mechanical strength, such as polyetherimide (PEI) or cyclic olefin copolymer (COC). Additionally, reducing the drawing temperature can help preserve the polymer’s viscosity throughout the thermal process. Another strategy involves lowering the surface tension *γ* by incorporating a secondary polymer into the preform (Supplementary Fig. S7). This adjustment alters the Laplace pressure to reflect the interfacial tension between the two polymers, potentially achieving a tension much lower than that of a single-polymer preform.

The second influencing force is the introduction of rotational force during the rotational drawing process in the horizontal plane, leading to deformations in the shapes of the microchannels (Supplementary Fig. S6c–f). The equation for angular momentum, *L*, provides insights into the role of rotary inertia in the thermal drawing process:

2 $$L=mvr.$$

Here, *m* signifies the mass of the polymer material encircling the channel, *r* is the radius of the channel situated in the preform, and *v* represents velocity, which is expressed as *v* = *ωr*, with *ω* denoting the angular velocity.

One noteworthy implication of Equation (2) is that angular momentum, *L*, increases towards the preform’s outer surface. Consequently, greater rotational forces affect the polymer mass situated further from the central axis, resulting in more pronounced channel deformations. Importantly, these forces act in a way that draws the channel inward, as depicted in Fig. 2f and (Supplementary Fig. S6c–f. Understanding these rotational forces enables better control over channel deformation, thus facilitating a higher level of precision in designing intricate microfluidic structures.

Through meticulous control of these factors, a top-down methodology for channel profile design can be adopted. Moreover, by leveraging these forces, channel geometries that defy conventional techniques, such as semi-circular and rhombus-shaped channels, can be created.

### Operation principles of the 3D spiral microfluidics within fibers

Figure 2 demonstrates the versatility of our design, showcasing diverse microchannel profiles and intricate spiral geometries in 3D spaces. The particle movements in our design result from a superposition of inertial lift forces and Dean drag forces.

**Fig. 3:**
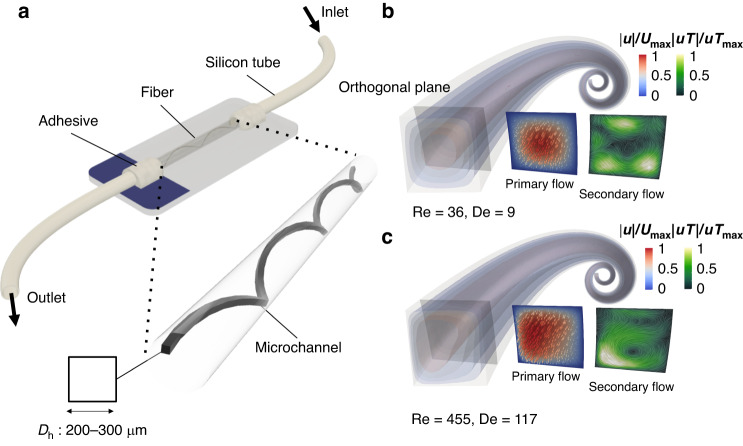
The microfluidic device-based fibers with 3D spiral channels and simulation study of its primary flow. **a** Schematics of the microfluidic device. The simulation revealed the shifting of the velocity profile of the primary flow towards the outer wall with *Re* = 36 in **b** and *Re* = 455 in **c**. A Dean vortex has emerged at a high *De* = 117

In inertial microfluidic devices operating at sufficiently high flow rates, inertial effects become significant. To quantitatively assess this, we calculated the Reynolds number (Re), defined as the ratio of inertial force to viscous force, given by

3 $$Re=\frac{\rho U{D}_{h}}{\mu },$$

where *U* denotes the average fluid velocity in the channel, and *D*_*h*_ represents the characteristic length scale of the microchannel, defined as *D*_*h*_ = 2*ab*/(*a* + *b*), with a and b being the length and width of the rectangular channel, respectively. For a square section, the hydraulic diameter *D*_*h*_ simplifies to the channel width (Fig. 3).

Our 3D spiral fiber devices feature inherent curvatures that give rise to unique microfluidic environments with climbing spirals. The channel curvature initiates a secondary, perpendicular Dean flow. This can lead to Dean vortices, which exert additional drag forces that impact particle positioning. The Dean number (De) quantifies this effect, defined as:

4 $$De=Re\sqrt{\frac{{D}_{h}}{d{\left(1+\frac{p}{\pi d}\right)}^{2}}},$$

where *D*_*h*_ is the microchannel width, *d* is the spiral diameter and *p* is the spiral pitch^28,29^.

Figure 3a presents the schematic of our fiber-based inertial microfluidic device. Numerical simulations were first carried out to understand the flow dynamics. Figure 3b and c display simulation results of the normalized velocity magnitudes of primary and secondary flows (**u** and **uT**) in a 3D spiral microchannel with a square cross-section. Isosurfaces of representative velocities near the outlet were plotted. Figure 3b reveals that at relatively low *Re* = 36 and *De* = 9, the flow is dominated by inertial lift forces, yielding a parabolic velocity profile characteristic of laminar flow. At higher *Re* = 455 and *De* = 117 (Fig. 3c), sufficient Dean flow is generated, altering the primary flow patterns. The emergence of a strong Dean vortex and the resulting changes in primary flow patterns generate additional Dean drag forces that re-equilibrate particle or cell positions within the flow. These forces are instrumental for efficient focusing and manipulation of particles or cells in 3D spiral microfluidics.

### Comparison of simulation and *μ*-TPIV Results

**Fig. 4:**
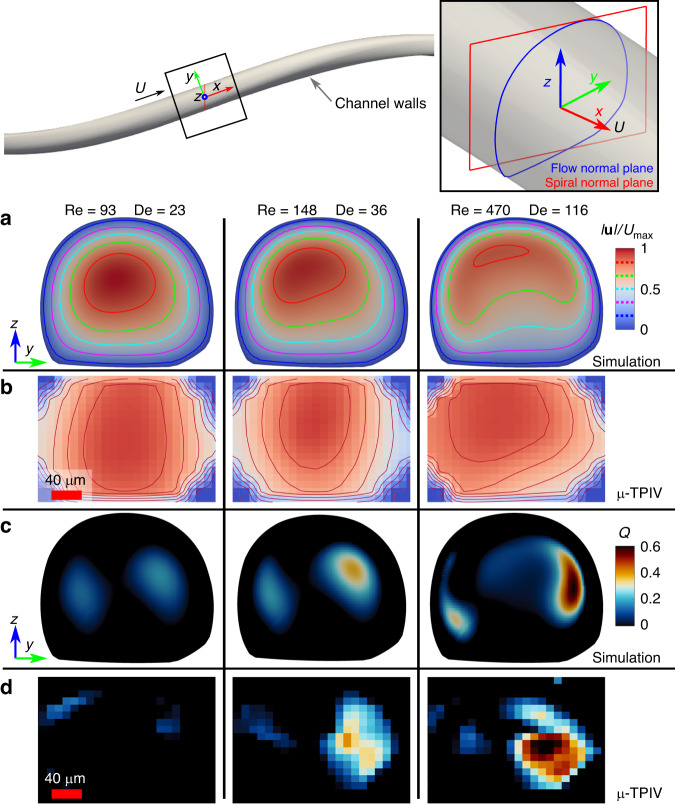
Comparison between simulation and *μ*-TPIV time-averaged measurements on flow-normal slices of *y-z*. **a**, **b** Normalized velocity magnitude ∣**u**∣/*U*_*max*_ and **c**, **d** *Q* criterion

We also performed *μ*-TPIV measurements on representative 3D spiral microfluidic fibers with the semi-circular microchannel shown in Fig. 2d. Six different flow rates were investigated (Table 1) and compared to the simulation results (Fig. 4 and Supplementary Figs. S8 and S9). We first defined a plane, orthogonal to the primary flow in order to evaluate the secondary flow (*y*-*z* in Fig. 4).

**Table 1:** flow parameters

case	flow rate (mL/min)	u (m/s)	Re	De
1	0.50	0.160	37	9
2	0.79	0.263	58	14
3	1.25	0.416	92	23
4	1.99	0.663	147	36
5	3.16	1.05	233	57
6	6.32	2.10	467	115

To identify and capture vortex regions within the flow, we employed the *Q* criterion^30^, which has been widely used in fluid dynamics analysis. The *Q* criterion is based on the eigenvalues of the velocity gradient tensor. It quantifies the local rotation and strain rates of the flow field. Regions with positive values of *Q* indicate regions dominated by rotational motion, suggesting the presence of vortices. Specifically, *Q* is the second invariant of ∇ **u**, written as $Q=\frac{1}{2}(| | \Omega | {| }^{2}-| | {{{\boldsymbol{D}}}}| {| }^{2})$, where Ω = ( ∇ **u** − (∇**u**)^⊺^)/2 and ***D*** = ( ∇ **u** + (∇**u**)^⊺^)/2 are the spin tensor and rate-of-strain tensor, respectively. By applying the *Q* criterion to our experimental data, we were able to identify and locate vortex regions within the 3D spiral microfluidic system^31^. These regions were characterized by higher values of *Q*, indicating the presence of swirling flow patterns and vortical structures.

Figure 4b–d presents three representative cases. At a low flow rate, characterized by *Re* = 93 and *De* = 23 (see Fig. 4a), the maximum flow velocity is observed along the channel center. A weak secondary flow pattern, corresponding to a small *De*, is noted in both the simulation and *μ*-TPIV results. Vortices are detected on both sides of the channel (see Fig. 4c, d). As the flow rates increase, leading to elevated *Re* and *De*, marked changes in the flow dynamics within the microchannel become apparent. Specifically, the region of maximum flow velocity migrates toward the outer wall of the microchannel. This shift can be attributed to the emergence of Dean flow, engendered by the microchannel’s spiral geometry. The Dean flow induces a secondary flow pattern perpendicular to the primary flow direction, which results in elevated flow velocity near the outer wall.

In addition to the alteration in maximum flow velocity, an evolution and amplification of vortices within the microchannel are observed. As both flow rates and the Reynolds number increase, these vortices become more distinct and stable. At *Re* = 470 and *De* = 116, one vortex, in particular, emerges as the dominant feature, displaying a stronger and more clearly defined rotational flow pattern.

The findings of our research underscore the successful generation of the Dean vortex within our fiber-based 3D spiral microfluidic devices. This Dean vortex serves a pivotal function in repositioning particles or cells within the fluid medium, thereby opening new avenues for particle manipulation and control. Employing *μ*-TPIV measurements, we were able to carry out an in-depth experimental assessment of flow dynamics within the microfluidic channels in 3D space. This sophisticated experimental approach yielded invaluable insights into the behavior of the Dean vortex and its influence on flow patterns. Additionally, our experimental outcomes exhibited a high degree of agreement with simulation data, corroborating the reliable and efficient formation of the Dean vortex. Such congruence between experimental and simulated results further substantiates the efficacy of our fiber-based 3D spiral microfluidics for generating and exploiting the Dean vortex for particle manipulation (see Supplementary Fig. S10).

Regarding future work, we intend to undertake further experiments and analyses to deepen our understanding of the relationship between secondary flow patterns, such as the Dean vortex, and the geometric variables of the microfluidic devices. This will encompass exploring the effects of channel profiles, spiral geometries, and other design parameters on the formation and behavior of the Dean vortex.

## Conclusion

We developed a rapid and efficient prototyping process, called the mini-rTDP, to manufacture 3D spiral fiber-based microfluidic devices with several key advantages. First, Mini-rTDP accommodates a broad spectrum of materials, offering considerable leeway in customizing device attributes such as chemical compatibility and optical clarity. Second, the methodology supports intricate device designs, including parallel channel configurations. This capability enables the integration of multifunctional microfluidic features within a single fiber device. Moreover, control over microchannel profiles, spiral radius, and pitch elevates the flexibility in crafting targeted fluid dynamics and particle manipulation functionalities.

We deployed both numerical simulations and a pioneering experimental imaging technique, *μ* TPIV, to evaluate flow dynamics. The identification of Dean vortices using the *Q* criterion, corroborated by computational simulations, underscores the potential of 3D spiral microfluidics in biomedical applications, particularly for particle and cell sorting. Future work will focus on refining spiral pitch to optimize Dean flow, a pivotal element in particle or cell repositioning. However, a trade-off exists between fine pitch and possible channel deformation due to the synergistic effects of thermal reflow and rotational forces.

The mini-rTDP process distinguishes itself by its rapidity and efficiency, offering a significant upgrade over traditional 2D lithography-based methods. Its scalability to industrial production enables the manufacturing of fibers extending over kilometers^25^. Such scalability is imperative for expediting R&D in microfluidics-related sectors, including biotechnology and diagnostics. Moreover, the simplicity and accessibility of mini-rTDP make it appealing to researchers and engineers. With minimal hardware requirements, it opens doors to novel material synthesis applications, such as the creation of internally twisted structures^32^. It also presents an innovative solution for lab-on-chip devices, enhancing point-of-care diagnostics^33^.

## Materials and methods

### Microfluidic fiber fabrication

For the preform material, a PC rod with a diameter of 15 mm was selected. PC is chosen for its optical clarity, which is beneficial for applications involving visualization or optical analysis within microfluidic channels. Additionally, PC has low surface interactions with fluorescent particles, minimizing any interference or undesired interactions between the material and the particles being manipulated or analyzed within the microfluidic system. First, we used a CNC milling machine (Magic F300) to machine grooves with predefined geometries in millimeter scale (Fig. 1a). Next, a PC film with a thickness of 100 μm (AS ONE 2-9587-26) was tightly wrapped around the machined PC rod, gradually building up the desired diameter. The end of the film was secured using heat-resistant tape to hold the preform in place. Subsequently, the preform was subjected to a consolidation process in a vacuum oven (AS ONE 1-2186-13) at a temperature of 185 °C and a vacuum level of 0.1 MPa for a duration of 40 min. To ensure a uniform consolidation, the orientation of the preform was reversed at the 20-min mark. This step was taken to ensure homogeneous consolidation throughout the preform, minimizing any variations in structure or properties.

In our mini-rTDP systems, we initially employed a linear guide mechanism to facilitate the drawing of the fiber’s preform (Supplementary Fig. S1). A holder, fitted with a DC motor, was mounted on the linear guide. The bait-off end of the preform was secured to this holder. By rotating the holder–and consequently the preform–during the thermal drawing process, we were able to twist and extend the inner channel of the preform, thereby creating spiral structures in 3D space (refer to Fig. 1c for illustration). To enhance precision and pave the way for large-scale production, we later developed an upgraded version of the mini-rTDP system. In this enhanced setup, the preform was fixed to a holder that could be rotated by a stepper motor with enhanced precision. Upon bait-off, the drawn fiber was collected using a capstan, which was controlled by a high-precision motor (Supplementary Fig. S2).

Before the rotational drawing process, the prepared preform was placed into our mini-rTDP with a customized electric furnace, and heated to a temperature of 260 °C, which was maintained for 20 min. This temperature is crucial for achieving the appropriate thermal conditions required for the subsequent drawing process (see Fig. 1b for visualization).

In our experiments, rotation speeds within the range of 50 to 100 rpm and vertical pulling speeds ranging from 10 to 30 mm/s were typically employed. These parameter ranges have been found to enable reliable prototyping and fabrication of spiral microfluidic fibers with pitches in the millimeter scale. By carefully selecting and adjusting these speeds, the desired pitch and structure of the spiral microfluidic fibers can be consistently achieved, ensuring reproducibility and control over the fabrication process.

In addition to PC, various other thermoplastics, such as highly transparent polymethyl methacrylate (PMMA) (Supplementary Fig. S11) and cyclic olefin COC, and thermoelastomers, for example, styrene-ethylene-butylene-styrene (SEBS) are also suitable for the rTDP process.

### Microfluidic device preparation

To characterize the flow dynamics within the 3D spiral microfluidics, a fiber-based microfluidic device was prepared using the following steps:

- Cutting and polishing: A section of the fiber, approximately 40 mm in length, was cut using a razor blade. Care was taken to ensure that the channel within the fiber was not collapsed during the cutting process. The cut end of the fiber was then carefully polished to streamline the flow. This polishing step helps minimize friction and disturbances during liquid injection.
- Connection and fixation: Both ends of the prepared fiber were connected to silicone tubing (MonotaRO MGJG-1*2) with an inner diameter of 1 mm. The connections were secured using a UV-curable epoxy (SEA FORCE 871040), ensuring a tight and reliable seal. The fiber, along with the tubing, was fixed onto a slide glass, providing stability for microscopic observation and analysis.
- Injection and visualization: A solution of deionized water (DI water) containing dyes was injected into the microfluidic device. This colored water served as a tracer to visualize the flow patterns within the spiral channel structures. The injection process allowed verification of the proper functioning of the device.

Microscopic observation of the device revealed representative spiral channel structures, as shown in Fig. 2c, e, and g. The colored water flowing through the channels demonstrated the successful formation and functionality of the 3D spiral microfluidic architecture within the fiber. This visualization technique provides insights into the flow dynamics and behavior within the microfluidic device, aiding in further analysis and characterization.

### Flow simulation

For detailed flow profile within the 3D spiral, we also performed numerical simulations using OpenFOAM^34^. To solve the mass (5) and continuity (6) equations

5 $$\rho \left(\frac{\partial {{{\bf{u}}}}}{\partial t}+{{{\bf{u}}}}\cdot \nabla {{{\bf{u}}}}\right)=-\nabla p+\mu {\nabla }^{2}{{{\bf{u}}}},$$

6 $$\nabla \cdot {{{\bf{u}}}}=0,$$

we assumed steady-state incompressible flow which can be solved by the conventional finite-volume solver SimpleFoam given values for *ρ* the fluid density and *μ* the dynamic viscosity of the fluid. These values are also used in the Reynolds number.

We modeled the sample geometry as a constant cross-section for two revolutions of the 3D spiral and scaled to a dimensionless hydraulic diameter *D*_*h*_ = 1 with a pitch of 70.4*D*_*h*_. We imposed a zero pressure condition at the outlet, uniform flow condition at the inlet, and the no-slip condition (***u*** = 0) at the channel walls. A hexahedral mesh was fit via the OpenFOAM snappyHexMesh utility using the simplified channel geometry and a uniform background mesh. Solutions were computed with a residual tolerance of 10^−7^ for velocity and pressure. Furthermore, a mesh convergence study was conducted at *Re* = 470 by measuring changes in the vorticity magnitude at the domain midpoint for successively finer meshes spanning 7.9 × 10^6^ − 83.4 × 10^6^ cells. The mesh used in this work had 41.2 × 10^6^ cells for a relative error below 1%.

### Microtomographic PIV

Flow within the channel was measured volumetrically using microtomographic particle image velocimetry (*μ*-TPIV)^35,36^. This approach is implemented using the LaVision FlowMaster system (LaVision, GmbH) comprised of a SteREO V20 stereomicroscope (Zeiss AG, Germany) mounted with dual cameras (Phantom VEO 410, 1280 x 800 pixels) imaging a volume of interest illuminated by a coaxial Nd:YLF laser (527 nm wavelength). The volume of interest was chosen to be where the channel is closest to the stereomicrocope, i.e. where the apparent surface curvature is minimized. Velocimetry is achieved by first reconstructing the three dimensional position of fluorescent particles suspended within the fluid (Fast Multiplicative algebraic Reconstruction Technique^37,38^), then deploying volume self-calibration (VSC)^39^) to refine their position. VSC with a high order (polynomial) model acts to further reduce image distortions due to imaging through the slight surface curvature^40^. In this study, the fluid was seeded with particles of 3 μm in diameter (excitation/emission 530 nm/607 nm, PS-FluoRed-Particles, Microparticles GmbH, Germany) to a particle concentration of approximately 0.03 particles per pixel at the desired magnification. Particle displacements over time (and thus the velocity field ***u***) are computed from a multi-grid iterative cross-correlation within a commercial PIV software (DaVis 10.1.2, LaVision GmbH), with a final pass at 32 × 32 × 32 voxels with 75% overlap. Flow was captured at a velocity-dependent frame rate such that no particle moved more than 8 pixels between frames for a sequence of 200 frames which was ultimately averaged for the velocity field **u**.

### Supplementary information

Supplementary Material
