# Supplementary material for: Twisted fiber microfluidics: a cutting-edge approach to 3D spiral devices
Source: Microsyst Nanoeng. 2024 Jan 22;10:14. doi: 10.1038/s41378-023-00642-9 (PMC10800335; doi:10.1038/s41378-023-00642-9)
Supplement: Supplementary file 1 — Supplementary Material [file 41378_2023_642_MOESM1_ESM.pdf]

Twisted Fiber Microfluidics: A Cutting-Edge Approach to 3D Spiral Devices

Shunsuke Kato[1], Daniel W. Carlson[2], Amy Q. Shen[2*], Yuanyuan Guo[3,4,5*]

[1]Department of Electrical, Information and Physics Engineering, School of Engineering, Tohoku University, Aoba-ku, Sendai, 980-8579, Miyagi, Japan.
[2*]Micro/Bio/Nanofluidics Unit, Okinawa Institute of Science and Technology, Onna, Kunigami-gun, 904-0495, Okinawa, Japan.
[3*]Frontier Research Institute for Interdisciplinary Sciences (FRIS), Tohoku University, Aoba-ku, Sendai, 980-0845, Miyagi, Japan.
[4]Graduate School of Biomedical Engineering, Tohoku University, Aoba-ku, Sendai, 980-8579, Miyagi, Japan.
[5]Department of Physiology, Graduate School of Medicine, Tohoku University, Aoba-ku, Sendai, 980-8575, Miyagi, Japan.

*Corresponding author(s). E-mail(s): amy.shen@oist.jp; yyuanguo@fris.tohoku.ac.jp;

List of Figures

S1 The schematics of the customized mini-rTDP system for prototyping spiral fibers. (a) The preform-to-fiber rotational and pull process. (b) The detailed diagram of the mini-rTDP system, showing its simple setup, similar to the pipette-pulling systems used in electrophysiology. 3

S2 Enhanced mini-rTDP system for refined control of fiber spirals and scalable production. (a) Schematic overview of the system. (b) Advanced control features in the updated rTDP system: rotation and capstan functionalities are managed by precision-engineered step motors. The down-feed process employs an exceptionally accurate linear guide. Furthermore, the heating component is tailored with a closed-loop temperature regulation system, capable of sustaining temperature fluctuations within a 1°C range. 3

S3 Tunable Cross-Sectional Profiles in Fiber-Based 3D Spiral Microfluidics. (a) Depicts a fiber cross-section with an off-centered oval microchannel, while (c) showcases a round microchannel. In (b) and (d), the side views of the fibers from (a) and (c) reveal the intricate spiral patterns. This versatility expands design possibilities beyond traditional square or rectangular profiles. 4
S4 The cross-section profile of the microchannel along the length of the microfluidic device based on spiral fibers. (a)-(e) show the cross section of fiber obtained at 5 mm intervals. The profile of the microfluidics is maintained, indicating the deformation or distortion of the microchannel shape is minimal. 4
S5 The mechanism of channel deformation. (a) Thermal reflow led to the rounding of corners. (b) The rotational force resulted in inward deformation of the channel. 5
S6 The relationship between channel geometry and pitch. (a) Cross-sectional view of a fiber spiral with an extended pitch. (b) Lateral perspective of the spiral from (a). (c) Cross-sectional view of a fiber spiral with a reduced pitch. (d) Lateral perspective of the spiral from (c). (e) Another cross-sectional depiction of a fiber spiral with a shortened pitch. (f) Side perspective of the spiral from (e). 5
S7 Polycarbonate and acrylonitrile butadiene styrene (ABS)-integrated fiber for mitigating channel deformation. (a) The schematics of the preform design. (b) Side view of the fiber spiral. (c, d, e, f) Cross-section view at three positions of 5 mm apart. 6
S8 Comparison between the simulations and µ-TPIV time-averaged measurements on flow-normal slices across a wide range of Re and De. (a, b) Normalized velocity magnitude $|\mathbf{u}|/U_{max}$. (c, d) Normalized secondary flow velocity magnitude $|\mathbf{u}|/U_{max}$. (e, f) Q criterion. 6
S9 Comparison between simulation and PIV measurements. (a) Representative cross-sectional view of the profile of microfluidics within the fiber. (b) Lateral perspective of the fiber spiral. (c, d) Normalized velocity magnitude of the primary flow. 7
S10 The potential inertial focusing within 3D spiral microfluidics. (a) The tomographic images of polystyrene particles close to the outlet (about 30 mm away from the inlet) under low Re and high Re. (b-c) The fluorescent intensity distribution across the z-axis of 2 µm diameter particles in (b) and 5 µm diameter particles in (c). The preliminary results reveal that the larger particles tend to migrate towards the outer wall, than the smaller ones at a higher Re and De. These results suggest the presence of centrifugal forces and potential particle-focusing effects within the 3D spiral microfluidic device. 8
S11 Polycarbonate and PMMA-based fiber and PEI-based fiber. (a) Diagram illustrating the preform preparation process. (b) Side view of the fiber spiral. (c)PEI-based preform (d) Side view of the PEI-based fiber spiral. (e) Cross-section view of the PEI-based fiber spiral with a single microchannel. (f) Cross-section view of the PEI-based fiber spiral with parallel microchannels. 9

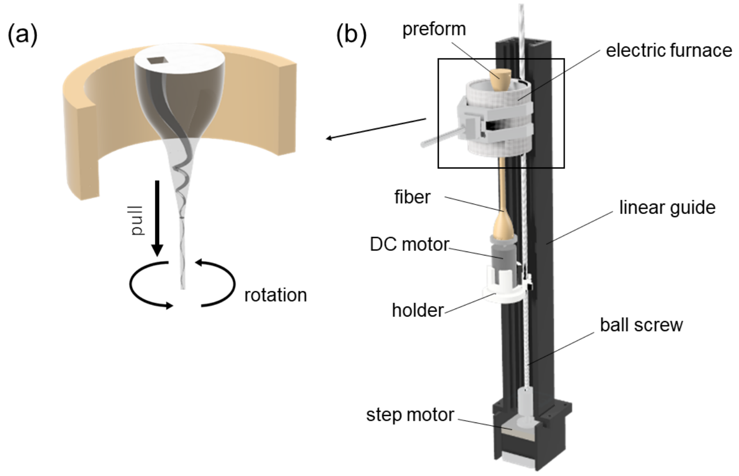

Fig. S1 The schematics of the customized mini-rTDP system for prototyping spiral fibers. (a) The preform-to-fiber rotational and pull process. (b) The detailed diagram of the mini-rTDP system, showing its simple setup, similar to the pipette-pulling systems used in electrophysiology.

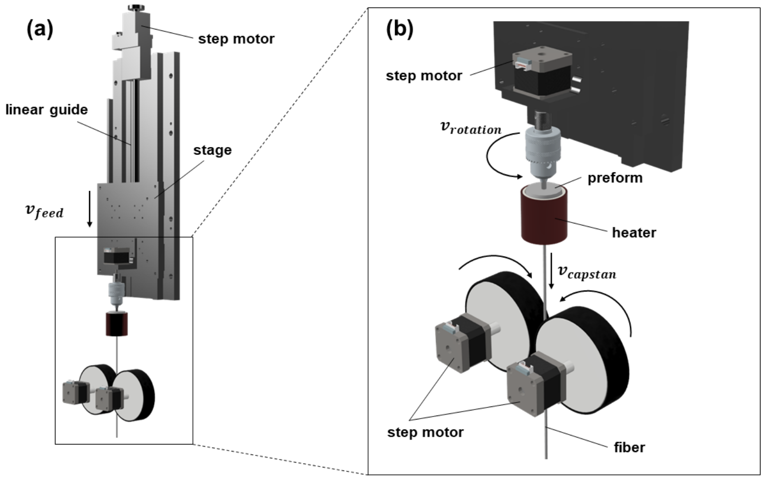

Fig. S2 Enhanced mini-rTDP system for refined control of fiber spirals and scalable production. (a) Schematic overview of the system. (b) Advanced control features in the updated rTDP system: rotation and capstan functionalities are managed by precision-engineered step motors. The down-feed process employs an exceptionally accurate linear guide. Furthermore, the heating component is tailored with a closed-loop temperature regulation system, capable of sustaining temperature fluctuations within a 1°C range.

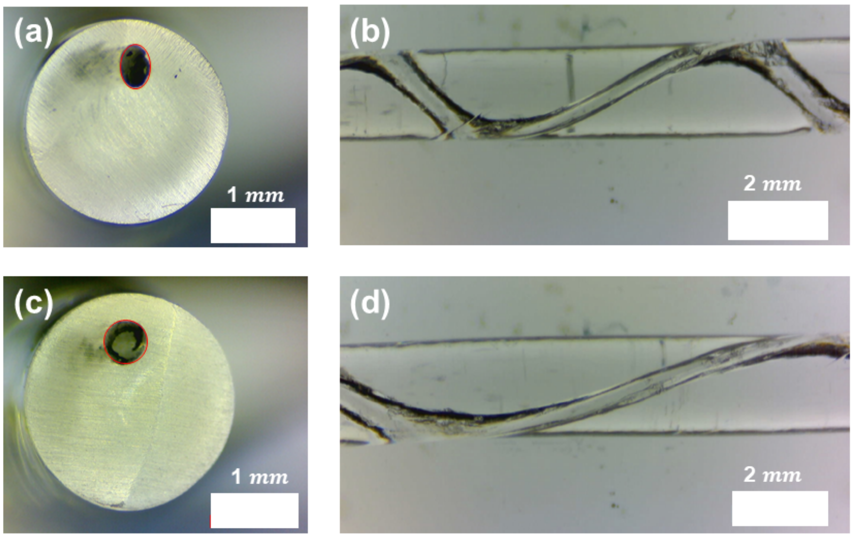

Fig. S3 Tunable Cross-Sectional Profiles in Fiber-Based 3D Spiral Microfluidics. (a) Depicts a fiber cross-section with an off-centered oval microchannel, while (c) showcases a round microchannel. In (b) and (d), the side views of the fibers from (a) and (c) reveal the intricate spiral patterns. This versatility expands design possibilities beyond traditional square or rectangular profiles.

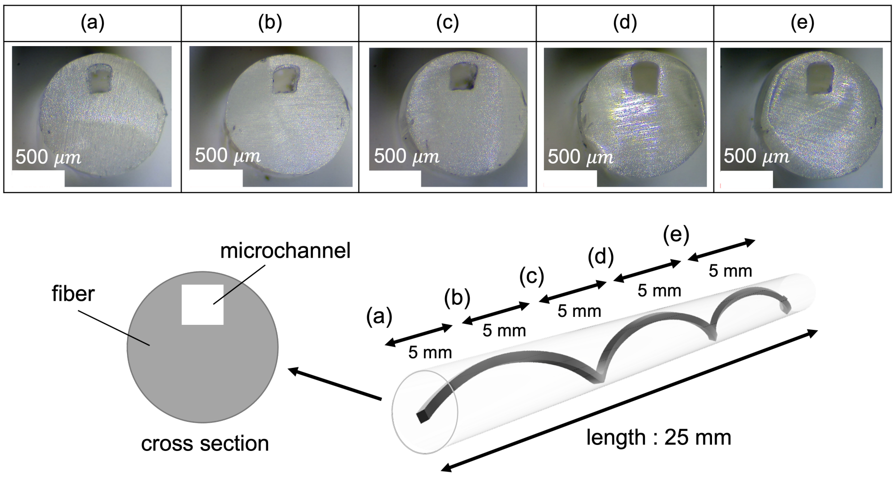

Fig. S4 The cross-section profile of the microchannel along the length of the microfluidic device based on spiral fibers. (a)-(e) show the cross section of fiber obtained at 5 mm intervals. The profile of the microfluidics is maintained, indicating the deformation or distortion of the microchannel shape is minimal.

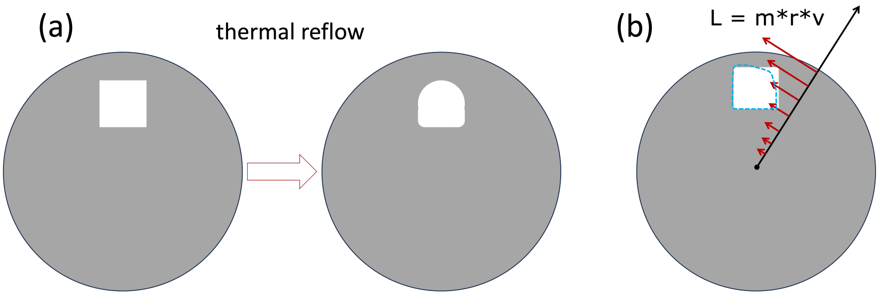

Fig. S5 The mechanism of channel deformation. (a) Thermal reflow led to the rounding of corners. (b) The rotational force resulted in inward deformation of the channel.

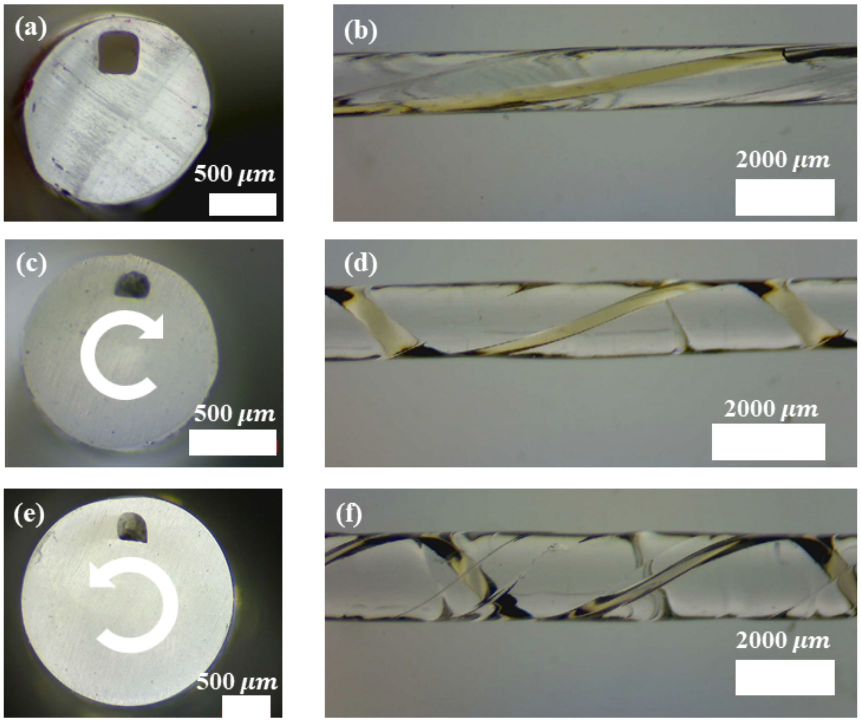

Fig. S6 The relationship between channel geometry and pitch. (a) Cross-sectional view of a fiber spiral with an extended pitch. (b) Lateral perspective of the spiral from (a). (c) Cross-sectional view of a fiber spiral with a reduced pitch. (d) Lateral perspective of the spiral from (c). (e) Another cross-sectional depiction of a fiber spiral with a shortened pitch. (f) Side perspective of the spiral from (e).

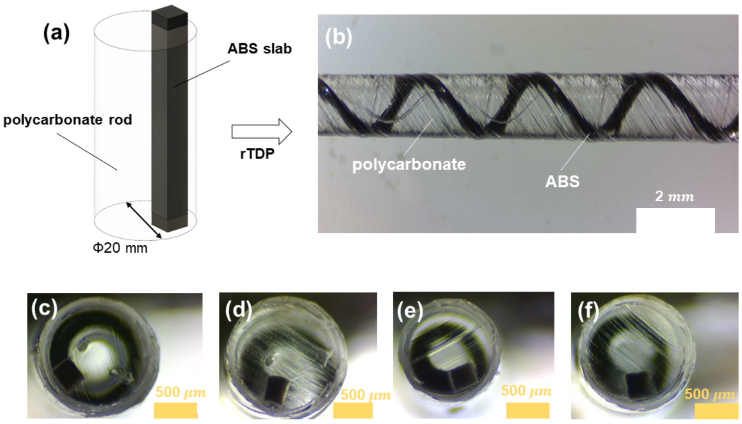

Fig. S7 Polycarbonate and acrylonitrile butadiene styrene (ABS)-integrated fiber for mitigating channel deformation. (a) The schematics of the preform design. (b) Side view of the fiber spiral. (c, d, e, f) Cross-section view at three positions of 5 mm apart.

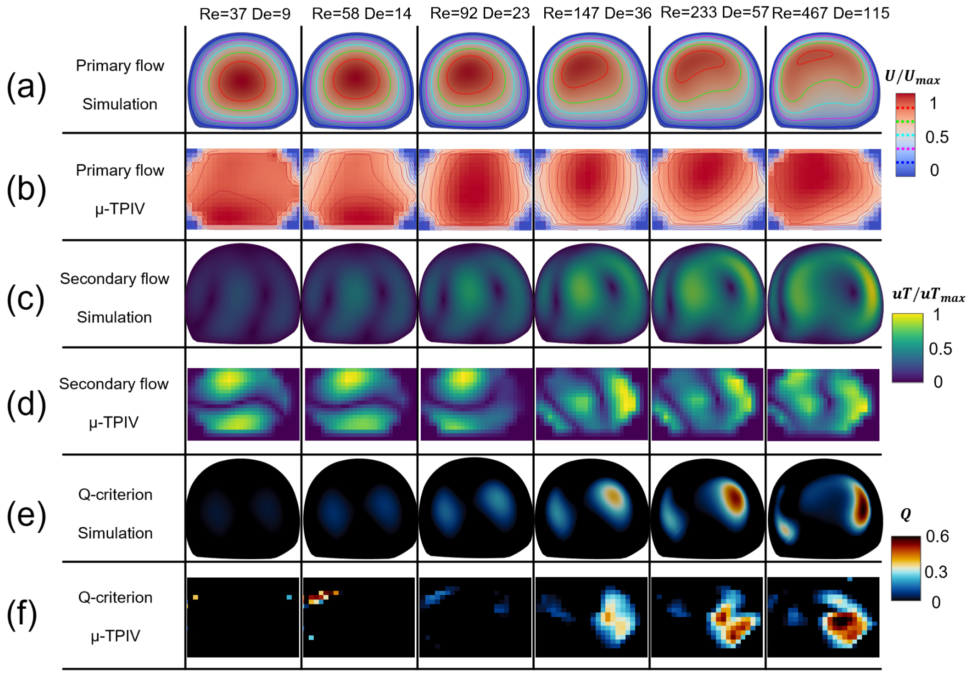

Fig. S8 Comparison between the simulations and μ-TPIV time-averaged measurements on flow-normal slices across a wide range of Re and De. (a, b) Normalized velocity magnitude $|\mathbf{u}|/U_{max}$. (c, d) Normalized secondary flow velocity magnitude $|\mathbf{u}|/U_{max}$. (e, f) Q criterion.

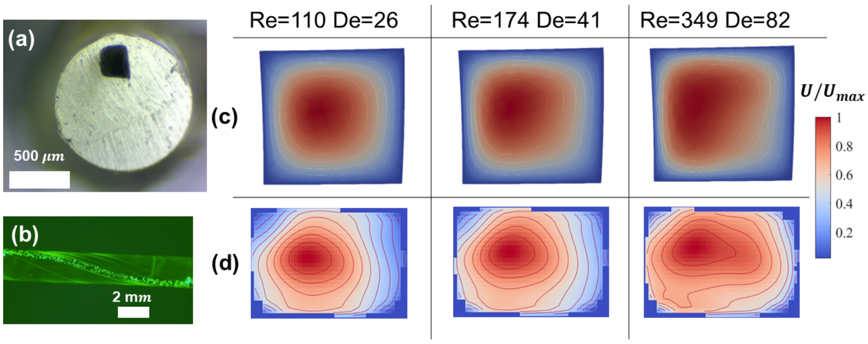

Fig. S9 Comparison between simulation and PIV measurements. (a) Representative cross-sectional view of the profile of microfluidics within the fiber. (b) Lateral perspective of the fiber spiral. (c, d) Normalized velocity magnitude of the primary flow.

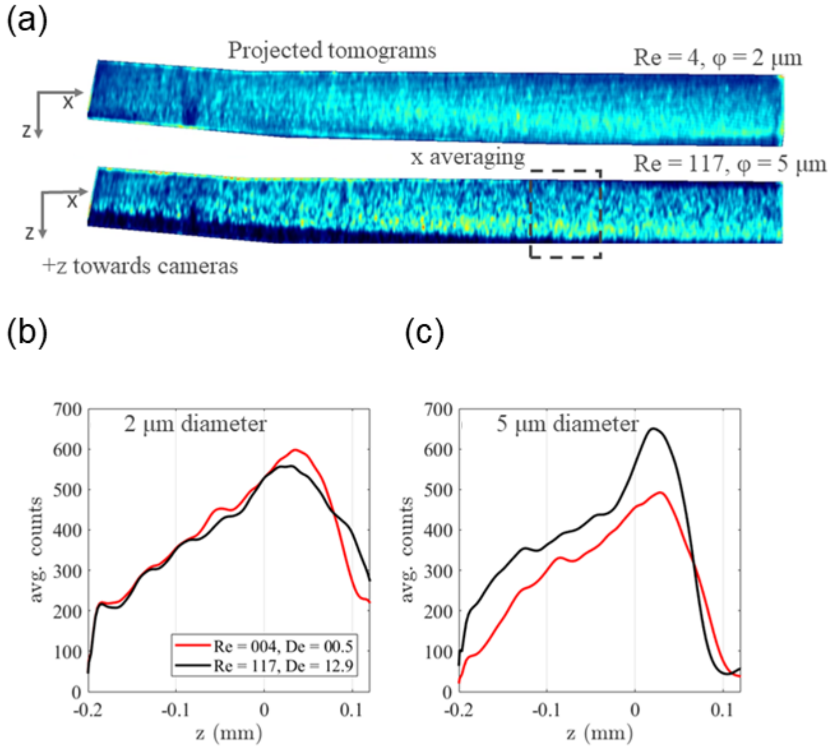

Fig. S10 The potential inertial focusing within 3D spiral microfluidics. (a) The tomographic images of polystyrene particles close to the outlet (about 30 mm away from the inlet) under low Re and high Re. (b-c) The fluorescent intensity distribution across the z-axis of 2 μm diameter particles in (b) and 5 μm diameter particles in (c). The preliminary results reveal that the larger particles tend to migrate towards the outer wall, than the smaller ones at a higher Re and De. These results suggest the presence of centrifugal forces and potential particle-focusing effects within the 3D spiral microfluidic device.

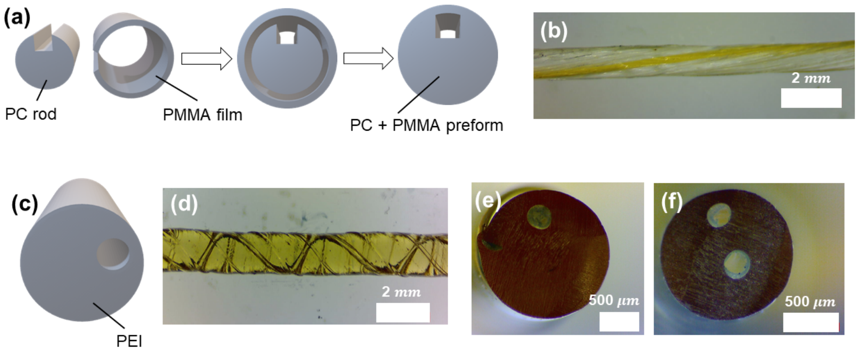

Fig. S11 Polycarbonate and PMMA-based fiber and PEI-based fiber. (a) Diagram illustrating the preform preparation process. (b) Side view of the fiber spiral. (c)PEI-based preform (d) Side view of the PEI-based fiber spiral. (e) Cross-section view of the PEI-based fiber spiral with a single microchannel. (f) Cross-section view of the PEI-based fiber spiral with parallel microchannels.
